# The Discrepancy Between Hemoglobin A1c and Glucose Management Indicators in 26 Patients Treated With Continuous Glucose Monitoring in an Internal Medicine Residency Clinic

**DOI:** 10.7759/cureus.56768

**Published:** 2024-03-23

**Authors:** Andre E Manov, Nathan Holt, Esar Dini, Ranier Rivera, Ashrita Donepudi, Rakahn Haddadin, Kyle Mefferd, Inam Qadir

**Affiliations:** 1 Internal Medicine, Sunrise Health Consortium Graduate Medical Education (GME), Las Vegas, USA; 2 Transitional Year, MountainView Hospital, Las Vegas, USA; 3 Internal Medicine, MountainView Hospital, Las Vegas, USA; 4 Research, MountainView Hospital, Las Vegas, USA

**Keywords:** continuous glucose monitoring (gmi), insulin, glucose management indicator (gmi), hba1c, diabetes mellitus

## Abstract

We conducted a retrospective observational cohort study between 2020 and 2023 in 26 patients with type 1 and type 2 diabetes mellitus (DM) who were using 3-4 injections per day of insulin and were monitored by continuous glucose monitoring (CGM). The goal of this retrospective observational cohort study is to compare these two metrics in an internal medicine community primary care residency clinic. We used CGM devices, Dexcom G6 and G7, and Freestyle Libre 3. The goal was to compare the patient’s hemoglobin A1c (HbA1c) taken during their clinic visit by phlebotomy as a marker for diabetic control with an estimated HbA1c glucose management indicator (GMI) derived from the 30-day CGM readings. HbA1c is derived from the blood, while the GMI value is derived from the interstitial fluid. Both parameters were taken within 30 days of each other. GMI was taken in the last 30 days. We excluded patients with known anemia, chronic kidney disease, polycythemia, cirrhosis of the liver, or metabolic dysfunction associated with steatohepatitis (MASH) because disease states can affect the measured HbA1c. Also, pregnant and African American patients were excluded. We concluded the measured HbA1c was 0.34% (4 mmol/mol) higher than the CGM-derived GMI. The relationship between factors that affect glycemic control was discussed in the article, as well as the future utilization of them in improving diabetic control and management. As the use of CGM continues to grow, addressing differences between laboratory-measured HbA1c and CGM-derived GMI is critical.

## Introduction

Reliable glucose measurements are crucial for effective diabetes management [1]. Laboratory-measured hemoglobin A1c (HbA1c) is the gold standard for assessing glycemic control in the last 2-3 months and measuring the effectiveness of diabetes therapy. HbA1c was first introduced into clinical practice in the early 1980s and has been shown in multiple studies to correlate with the risk of developing long-term microvascular and macrovascular complications. HbA1c is the glycated percentage of hemoglobin in circulating red blood cells (RBC) during the previous 2-3 months [1]. 

Implementing CGM in diabetes care is the most important advancement in diabetology in the last 20 years. Real-time data from the CGM empowers patients and their healthcare providers to make informed decisions on diet, physical activity, and medication adjustment, which improves glycemic control compared to self-monitoring blood glucose (SMBG). Studies reveal CGM's superiority over SMBG, with improved glucose control and reduced variability, applicable to both type 1 and insulin-requiring type 2 diabetes [2]. Utilizing an implantable device, CGM tracks the glucose concentrations in the body’s interstitial fluid every 1-5 minutes, 24 hours a day, allowing clinicians and patients with diabetes to optimize their management. The mean glucose concentration from CGM is used to derive the glucose management indicator (GMI) to approximate the estimated HbA1c using a population-based regression formula [2]. The 14-day GMI usually correlates highly and positively with the HbA1c checked within the last month [3]. 

Understandably, clinicians and patients with diabetes are often confused or disappointed when the GMI and laboratory measured A1C do not match. The original description of GMI in 2017 reported differences in calculated GMI with measured HbA1c. This study looked at 528 individuals without other comorbidities from four clinical trials [2]. It was recognized that GMI and laboratory measured HbA1c may differ due to several non-glycemic factors involved in each calculation [4]. 

Recent studies also showed a clinically significant discordance of ≥0.5% (6 mmol/mol) between HbA1c and GMI in 36-43% of the 144 patients participating in the hyperglycemic profiles in obstructive sleep apnea (HYPNOS) trial [5]. A possible explanation involves factors that affect RBC turnover, such as hemolytic anemia, iron deficiency anemia, polycythemia, and B12 deficiency anemia, as well as pregnancy or glycation kinetics due to genetics, the presence of advanced chronic kidney disease, metabolic dysfunction-associated steatohepatitis (MASH), and cirrhosis of the liver and race, among others [5]. 

Clinicians need to be aware that HbA1c may not be as accurate a reflection of mean glucose as previously thought. The relationship between mean glucose and HbA1c needs to be interpreted for each patient on an individual basis. It is essential that clinicians consider CGM metrics as a powerful alternative assessment of glycemic control [6]. 

As the use of CGM continues to grow, addressing differences between laboratory-measured HbA1c and CGM-derived GMI is critical [6]. The purpose of this retrospective observational study is to compare these two metrics in an internal medicine residency clinic. 

## Materials and methods

This study was approved by our institutional review board at MountainView Hospital. We performed a retrospective observational study of 26 patients in our internal medicine clinic with type-1 DM and type-2 DM who used CGM in the last three years, from 2020 to 2023. We performed a sequential review of patients who were prescribed insulin injections and were using CGM at least 90% of the time seen at our clinic. All of our patients were using three to four injections of basal insulin once a day and bolus rapid-acting insulin before meals 2-3 times a day. We used the CGM devices Dexcom G6 and G7 (Dexcom, Inc., San Diego, California) and Freestyle Libre 3 (Abbott, Alameda, California). 

We required that patients have a documented HbA1c value measured by high-performance liquid chromatography no more than 30 days before or after the associated office visit and CGM upload. The uploaded data contained a 30-day CGM reading with an estimated GMI. Laboratory tests used to identify anemia, polycythemia, and chronic kidney disease (CKD) were drawn less than 12 months from the incident HbA1c and CGM-derived GMI. To exclude metabolic dysfunction-associated steatohepatitis (MASH) and cirrhosis of the liver, we used ultrasound elastography in high-risk patients as determined by the Fibrosis-4 (Fib-4) Index for Liver Fibrosis. Cirrhosis was excluded in patients who were using alcohol or had other risk factors for liver disease by checking their laboratory parameters and obtaining an ultrasound of their liver and spleen. We used the device's estimated HbA1c (GMI).

We monitored and extracted qualifying encounters from the eClinicalWorks electronic medical record system, which had over 1000 patients. A total of 26 patients were analyzed in this study, but we screened 40 patients. We excluded 14 patients based on our exclusion criteria. Patients with anemia, polycythemia, chronic kidney disease, cirrhosis of the liver, MASH, African Americans, and pregnant women were excluded from the study. These diseases, African American race, or pregnancy state, as discussed above, could alter HbA1c, which would affect the comparison between HbA1c and GMI. The patients were followed closely in our clinic, with follow-up phone calls every two weeks and in-office visits every 2-3 months. 

Within-patient HbA1c and GMI values were compared by analyzing data obtained within 30 days of each other. The GMI value was calculated as an average of 30 days either before or after the HbA1c measurement. We chose to use 30 days of CGM data to generate the most optimized CGM metrics in terms of glucose variability. Recent studies on optimal sample duration for CGM demonstrated that 10-14 (24) days of CGM data were sufficient to accurately reflect three months of glycemic control measured by checking HbA1c obtained from blood [4-6]. Another requirement was that the CGM report had to contain at least 90% of the days (27 of 30 days) of sensor usage. HbA1c was measured from the phlebotomy blood draw done in our clinic and was found in all of our patients between 7 and 11%, with a mean of 8.7%. GMI was measured by the following CGM devices: Dexcom G6, G7, or Freestyle Libre 3. The inclusion and exclusion criteria used are described in Table 1. 

**Table 1:** Inclusion and exclusion criteria

Inclusion Criteria	Exclusion Criteria
Patients ages 18-90 years old	Patients who were non-compliant with dietary and exercise recommendations. Patients with chronic kidney disease, anemia, MASH, cirrhosis of the liver and polycythemia and being African-American.
Patients with the diagnosis of Type 1or type 2 diabetes mellitus	Patients who were unable to understand the instructions for the titration of insulin-based on their CGM data.
Patients with HbA1c > 7% (53 mmol/mol) and who were receiving their primary care in the internal medicine residency clinic	Patients wearing their CGM device less than 70% of the time.
Patients with uncontrolled blood glucose levels while using SMBG for equal or greater than four times daily	Patients with impaired decision-making capacity.
Patients receive their primary care only at the internal medicine residency clinic	Patients missing >2 scheduled visits.
Patients can use a CGM device	Patients who are pregnant or incarcerated.
Patients on 3-4 injections of Insulin +/- oral diabetic medications and using CGM at least 90% of the time	Patients unresponsive to calls from the clinic.
The patient can adjust their Insulin based on the CGM data	Patients whose insurance did not cover the CGM device.

## Results

**Figure 1:**
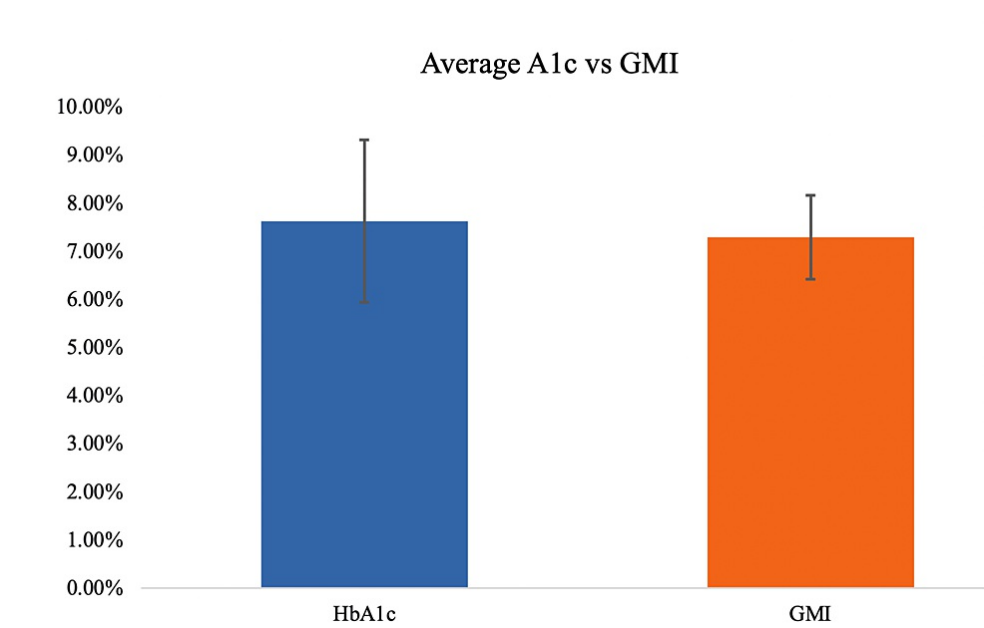
Depiction of average differences between A1c and GMI (estimated A1c)

To assess if this difference between blood-derived HbA1c and CGM derived from the interstitial fluid GMI (HbA1c) was significant, as a part of our interpretation of the results and statistical analysis, a paired samples t-test was conducted. Specifically, we compared the means of laboratory-measured HbA1c and CGM-derived GMI (predicted HbA1c) to illustrate any differences. Two outliers were detected as assessed by inspection of a boxplot for values greater than 1.5 box lengths from the edge of the box. However, these outliers were kept within the sample data because the data was revealed to not meet the assumption of normality as revealed by a significant (p<.001) Shapiro-Wilk's test and as assessed by visual inspection of the normal Q-Q plot. However, given that t-tests are fairly robust to violations of normality, we chose to run the paired sample t-test. As seen in Figure 1, in the 26 patients with type-1 DM and type-2 DM, the HbA1c was, on average, 0.34% (4 mmol/mol) higher than the estimated HbA1c (GMI) derived from the 30-day CGM data. However, the paired t-test revealed no significant difference was observed between the HbA1c (7.7 ± 1.6%) (61 mmol/mol ±17 mmol/mol) and GMI (7.3 ± 0.0.88) (mmol/mol± 10 mmol/mol), t (25) = 1.625, p =.117, 95% CI [-.12, 1.04]. Our results of the HbA1c and CGM-derived GMI are depicted in Figure 1.

One method of checking HbA1c was by measuring the level in the blood, and the other was to estimate HbA1c using the data from CGM. Notwithstanding, the HbA1c measured was numerically higher than the GMI, which was CGM-derived, although both values were non-statistically significantly different. 

In 53% of the patients in our retrospective study, the HbA1c was higher than the GMI. When comparing the two parameters, we found that in 43% of the patients, GMI was higher than HbA1c, and in 3.8%, those two parameters were equal. The difference between HbA1c and GMI was less than 0.1% (1 mmol/mol) in 3.85% of the patients and between 0.1 and 0.49% (1-5 mmol/mol) in 26.9% of the studied patients. In 50% of the studied patients, the difference was between 0.5 and 1% (5-11 mmol/mol), and in 19.2% of our patients, the difference between HbA1c and GMI was more than 1% (11 mmol/mol). 

**Figure 2:**
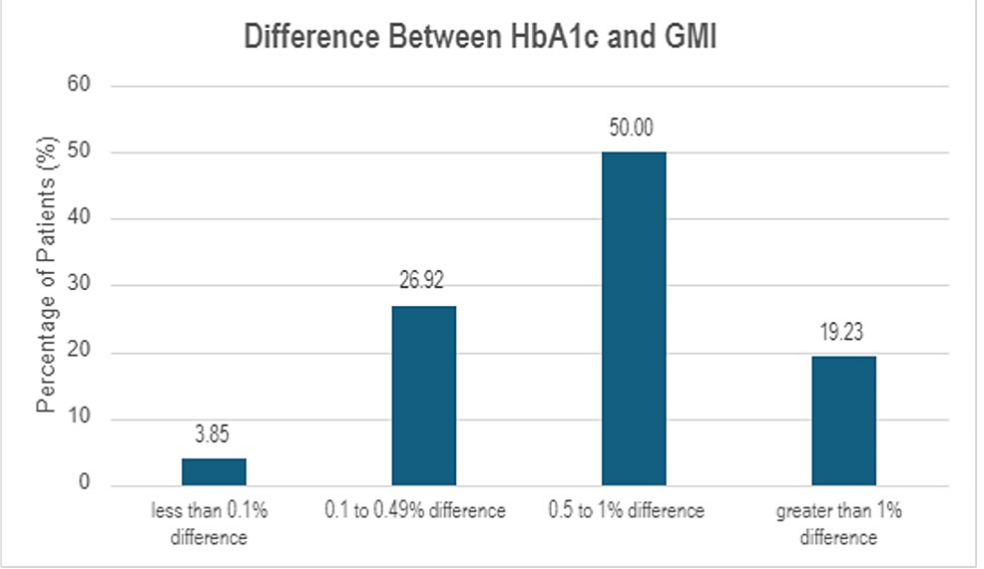
Percentage difference between HbA1c and GMI

The differences between those two parameters of glucose control could not have been explained, as discussed above, by the factors that most often increase or decrease falsely HbA1c as well as race. In a study of only Caucasian and Asian patients, our results of the percentage difference between GbA1c and CGM-derived GMI are depicted in Figure 2.

## Discussion

Continuous glucose monitoring provides a more comprehensive and detailed view of blood glucose levels, making it a valuable tool for diabetes management in both Type 1 and Type 2 [3,7-11]. HbA1c has traditionally been the standard measure of mean glucose levels in the last 2-3 months in the population, but as we know from the literature, sometimes it can be misleading [12]. GMI is measured from the glucose in the interstitial fluid, while A1c is measured from glucose attached to hemoglobin. Our retrospective observational study investigated the discrepancy between GMI and HbA1c in patients with type-1 DM and type-2 DM while using CGM. To our knowledge, no other studies on CGM have investigated the discrepancy between HbA1c and CGM-derived GMI by specifically using a range of 30 days of CGM data to assess glycemic control [8]. The numerical difference between the two parameters ensues from measuring HbA1c from the blood and CGM-derived GMI (HbA1c) from the interstitial fluid.

The major strength of our study is that we included the population with similar starting HbA1c ranges who were on 3-4 injections of insulin per day. Another strength of the study was that we compared the HbA1c derived from their CGM to the blood derived HbA1c taken within 30 days of each other, e.g., in similar populations of patients. To decrease the confounders and biases of the study, we used patients with similar baseline ranges of HbA1c and insulin injections per day. The other novel approach was that, compared to other studies on CGM-derived and blood-derived HbA1c, the CGM-derived values of GbA1c were not from 14-24 days but from 30 days. Also, a new approach was that the analysis of the study results included detailed descriptions of the numerical difference between the two parameters in exact percentages. The other novel thing about the study is that it was conducted in an internal medicine residency clinic and not in a specialized endocrine or primary care clinic.

Our study indicated a non-statistically significant difference between HbA1c and GMI. This is important to reassure patients as well as some healthcare organizations (for example, Medicare does not acknowledge the GMI) and medical providers who believe only blood-derived HbA1c is a valid measure of diabetes mellitus control.

The study revealed diverse numerical values, with 53.8% of individuals demonstrating a lower GMI value than HbA1c, while 42.3% exhibited an increased value. Only one of the participants had identical values for HbA1c and GMI. This can again be explained by the different body fluids from which the two parameters were measured. 

This study did not show the discrepancy between measured HbA1c from the blood and estimated HBa1C (GMI), which was CGM-derived. 

We need to acknowledge that certain biases and confounders might have influenced our results. Our study has several limitations. This is just one study, and it was a retrospective observational cohort study. For example, if a medication change was made at the visit and the HbA1c was drawn a month later, that would have caused the results to be different from the CGM report. A perfect comparison would require a GMI and A1C on the same day with 60-90 days of CGM data. Based on our experience and other studies, although this can be the perfect scenario, as discussed before, we do not believe this would have significantly changed the differences between HbA1c and CGM-derived GMI (HbA1c). Additionally, three types of CGM devices were used. While Dexcom G7 was the predominant CGM device used by our patients, our study included CGM devices from Dexcom G6 and Freestyle Libre 3. This could have influenced our research because each device has different proprietary technology and different metrics for calibration. The study population was mostly Caucasians, excluding African Americans, who, on average, have higher HbA1c [13]. Another bias of the study is that we have excluded patients with anemia, chronic kidney disease, cirrhosis of the liver, polycythemia, and MASH. Another confounder of the study might have been the different antidiabetic medications besides insulin the patients were using. This decreases the validity and generalizability of our study. The sample size was small-26 patients. HbA1c was taken at approximately every three months of follow-up in our clinic. However, as GMI was based on continuous measurement. There was up to a 30-day difference from the time HbA1c was measured, although we do not believe this could have significantly changed our results. 

Another limitation of the study was that for our patients’ population, dietary and weight loss counseling was given at each clinic visit, but patients’ diet and lifestyle habits were not closely monitored. This is in line with Bergenstal et al.’s meta-analysis, which found that GMI and HbA1c may differ because of non-glycemic factors [2]. 

Since laboratory-measured HbA1c and CGM-derived GMI may be used in different ways to inform patients about the glucose management plan, it is wise to discuss with patients both the laboratory-measured HbA1c target and the goals for glucose management based on CGM metrics and estimate from them HbA1c (GMI). We have respected those two parameters as different because HbA1c obtains measures for assessing glucose control from the blood, and GMI (HbA1c obtained from the CGM) obtains this measure from the interstitial fluid. The importance of the study was that those two parameters obtained from two different body fluids are not statistically significantly different. The mean blood glucose using CGM was 162.7 mg/dl (9.03 mmol/l), which was not statistically significantly different from the mean glucose obtained from the blood using HbA1c, which was 172.5 mg/dl (9.57 mmol/l). The advantage of monitoring the GMI is that it is not influenced by factors or medications that might affect HbA1c. Having real-time CGM glucose data and retrospective glucose pattern availability provides additional information to help guide appropriate medication or lifestyle selection and adjustment in real-time.

With the next phase of studies, we can increase the sample size and power of our study to establish a more detailed discrepancy between HbA1c and GMI with further control of the potential confounding factors discussed above. Our study demonstrates a small but non-significant difference between HbA1c and GMI, which can help in the future to more precisely individualize the insulin management of patients with diabetes mellitus. 

## Conclusions

In this study, we have explored the discrepancy between HbA1c (glycated hemoglobin) and GMI (Glucose Management Indicator) in a cohort of 26 patients undergoing glucose monitoring with CGM. However, the findings from this retrospective observational cohort study did not show any statistically significant difference between measured HbA1c and estimated HbA1c (GMI) derived from the CGM. This is important because some health care organizations use only HbA1c obtained from the blood of patients to measure the control of diabetes mellitus for healthcare and reporting purposes and do not incorporate GMI-CGM-derived HbA1c in assessing the control of diabetes. We have found a numerically higher HbA1c compared to the GMI. In conclusion, our study highlights the importance of considering both HbA1c and GMI in diabetes management, especially for individuals utilizing CGM.
